# The Use of Dynamic Contrast-Enhanced Magnetic Resonance Imaging for the Evaluation of Blood-Brain Barrier Disruption in Traumatic Brain Injury: What Is the Evidence?

**DOI:** 10.3390/brainsci11060775

**Published:** 2021-06-11

**Authors:** Sung Suk Oh, Eun-Hee Lee, Jong-Hoon Kim, Young Beom Seo, Yoo Jin Choo, Juyoung Park, Min Cheol Chang

**Affiliations:** 1 Medical Interdisciplinary Team, Medical Device Development Center, Daegu-Gyeongbuk Medical Innovation Foundation, Daegu 41061, Korea; ssoh@dgmif.re.kr (S.S.O.); ehlee@dgmif.re.kr (E.-H.L.); chooyj@dgmif.re.kr (Y.J.C.); 2 Department of Neurosurgery, College of Medicine, Yeungnam University, Daegu 41061, Korea; kjhns@yu.ac.kr (J.-H.K.); nsybseo@gmail.com (Y.B.S.); 3 Department of Rehabilitation Medicine, College of Medicine, Yeungnam University, Daegu 41061, Korea

**Keywords:** traumatic brain injury, dynamic contrast-enhanced magnetic resonance imaging, blood brain barrier, review

## Abstract

(1) Background: Blood brain barrier (BBB) disruption following traumatic brain injury (TBI) results in a secondary injury by facilitating the entry of neurotoxins to the brain parenchyma without filtration. In the current paper, we aimed to review previous dynamic contrast-enhanced magnetic resonance imaging (DCE-MRI) studies to evaluate the occurrence of BBB disruption after TBI. (2) Methods: In electronic databases (PubMed, Scopus, Embase, and the Cochrane Library), we searched for the following keywords: dynamic contrast-enhanced OR DCE AND brain injury. We included studies in which BBB disruption was evaluated in patients with TBI using DCE-MRI. (3) Results: Four articles were included in this review. To assess BBB disruption, linear fit, Tofts, extended Tofts, or Patlak models were used. $K^{Trans}$ and $v_{e}$ were increased, and the values of $v_{p}$ were decreased in the cerebral cortex and predilection sites for diffusion axonal injury. These findings are indicative of BBB disruption following TBI. (4) Conclusions: Our analysis supports the possibility of utilizing DCE-MRI for the detection of BBB disruption following TBI.

## 1. Introduction

Traumatic brain injury (TBI) is a common neurological disorder causing various disabilities, such as motor and sensory dysfunction, cognitive impairment and language problems [1]. Poor outcomes associated with TBI result from significant primary damage to the brain and subsequent secondary mechanisms of brain injury [2]. The blood-brain barrier (BBB) plays an important role in maintaining optimal brain function by regulating the interstitial fluid microenvironment [3]. BBB disruption is recognized as one of the common consequences of TBI [4,5]. BBB disruption leads to its failure to sufficiently block the transport of neurotoxins to the central nervous system (CNS) by separating the peripheral circulation from the CNS [4,5]. In addition, BBB damage results in cerebral edema. Accurate evaluation of the presence and degree of BBB disruption would be helpful for elucidating appropriate protocols for TBI treatment. However, no standardized evaluation tools exist for the assessment of BBB disruption after TBI.

Dynamic contrast-enhanced magnetic resonance imaging (DCE-MRI) is a noninvasive perfusion MRI technique that enables the evaluation of damage to the microcirculatory structure and dysfunction of the BBB [6]. To date, several studies have used DCE-MRI to detect BBB disruption in various neurological disorders, including stroke, brain tumor, dementia and mild cognitive impairment [7,8,9,10]. BBB disruption after TBI has also been evaluated in previous studies using DCE-MRI [11,12,13,14]. The present paper aimed to review these DCE-MRI studies and explore their utility for assessing BBB disruption following TBI. In addition, prior to reviewing available previous studies, we reviewed the parameters used in DCE-MRI studies for the analysis of BBB disruption.

## 2. Methods

We searched for studies that had analyzed BBB disruption following TBI using DCE-MRI. To this end, we analyzed the PubMed, Scopus, Embase and Cochrane Library databases for relevant studies published between January 1980 and May 2021. The key search phrase for identifying potentially relevant articles was dynamic contrast-enhanced OR DCE AND brain injury. The following inclusion criteria were applied during the selection of articles: (1) studies involving patients with TBI and (2) studies in which BBB disruption was evaluated using DCE-MRI. This review was limited to studies involving humans with TBI. Review articles and articles not written in English were excluded.

## 3. Results

### 3.1. Study Selection

**Figure 1:**
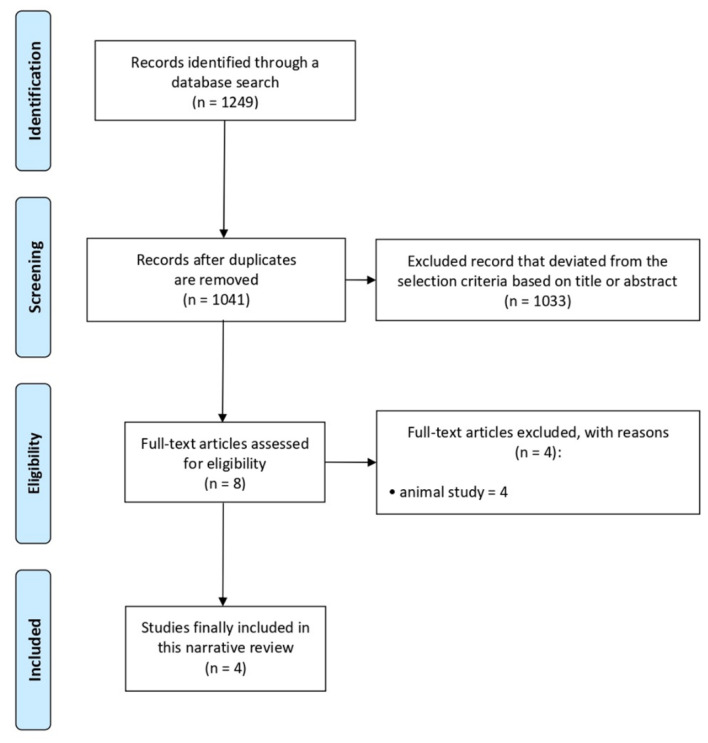
Flow chart of the selection process of relevant publications.

A total of 1249 potentially relevant articles were identified (Figure 1). We excluded 208 duplicate studies and excluded an additional 1041 publications after their titles and abstracts were reviewed. The full texts of the remaining eight studies were thoroughly assessed. Eventually, four studies were included in the review [11,12,13,14] (Table 1).

### 3.2. Parameters Used in DCE-MRI Studies

For the acquisition of DCE-MRI data in patients with TBI, a 3D Spoiled Gradient Echo (SPGR) sequence was utilized, and the imaging parameters were in-plane resolution (0.5–1 mm), slice thickness (3–5 mm), flip angle (6–20°), time resolution (6.5–7.7 s/scan), and total time (5–8 min).

**Figure 2:**
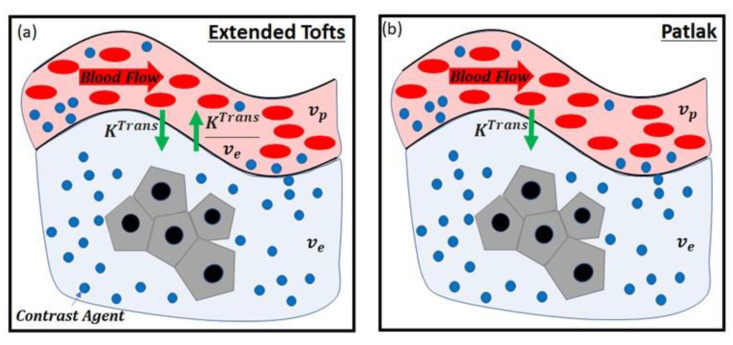
Pharmacokinetic models: (**a**) extended Tofts model, (**b**) Patlak model. $K^{Trans}$, volume transfer constant; $v_{e}$, extravascular extracellular space volume fraction; $v_{p}$, blood plasma volume fraction.

The models for assessing the disruption of the BBB included linear fit [11], Tofts [15], extended Tofts [16], or the Patlak model [17] (Table 2). In the linear fit model, the slope of the contrast agent concentration over the total acquisition time of DCE-MRI was calculated. The other methods, i.e., Tofts, extended Tofts, and Patlak models, are based on the pharmacokinetic models listed in Table 2. In each model, the BBB leakage via passive diffusion due to the concentration gradient between blood plasma and extracellular extravascular space (EES) after intravenous injection of a contrast agent is represented. As shown in Figure 2, the physiological compartments and parameters related to the BBB disruption were considered in each model. The meaningful parameters in the models for assessing the BBB disruption were $K^{Trans}$, $v_{p}$ and $v_{e}$, which indicate volume transfer contrast from the plasma into the EES across the vessel wall, blood plasma volume fraction, and EES volume fraction, respectively. Accordingly, the BBB disruption increases the values of $K^{Trans}$ and $v_{e}$, and decreases the value of $v_{p}$.

### 3.3. Review of Previous Studies

The first study on the usefulness of DCE-MRI in evaluating the BBB disruption following TBI was reported by Winter et al. in 2015 [12]. The authors prospectively recruited 14 patients with TBI. Of these patients, seven had mild TBI (Glasgow Coma Scale [GCS] 13–15), four patients had moderate TBI (GCS 9–12), and three patients had severe TBI (GCS 3–8). Winter et al. conducted DCE-MRI in all 14 patients at four to 30 days (mean, 11 days) after the TBI onset. In the T1 diffusion image of each patient, the $K^{Trans}$ was modified as the index over the BBB-disrupted area, reported in ml/100 gmintue^−1^ and assuming brain tissue density of 1 g/mL. The mean value of $K^{Trans}$ was 18.59 ± 17.28 mL/minute (range, 0.01–52.36 mL/minute). As the head injury after trauma became more severe, the $K^{Trans}$ value increased: 14.05 ± 16.33 mL/minute in mild TBI, 18.94 ± 14.46 mL/minute in moderate TBI, and 28.68 ± 24.55 mL/minute in severe TBI. However, a significant difference in $K^{Trans}$ value following the severity of TBI was not observed (*p* = 0.12) in that study. The authors also evaluated the presence of a significant correlation between $K^{Trans}\;$values and the results of single-photon emission computed tomography (SPECT) and serum S100B (small oligomeric cytoplasmic calcium-binding protein) levels. SPECT was conducted in eight out of 14 recruited patients, and the standardized uptake value (SUV) was measured. SUV reflects the metabolic rate of the brain microenvironment, and higher SUV values indicate that the brain injury caused by trauma is more severe. The SUV in the area of BBB disruption was calculated as 61.47 ± 77.18, 64.5 ± 69.03, and 154.38 ± 62.21 in patients with mild, moderate and severe TBI, respectively. However, despite the tendency of the SUV to be higher as the TBI was more severe, no significant difference was observed (*p* = 0.10). The $K^{Trans}$ value showed a significant positive correlation with the SUV. In addition, Winter et al. measured the blood level of S100B, which is found predominantly in astrocytes. Levels of S100B in the cerebrospinal fluid after TBI are typically 10 to 100 times higher than those in the serum. The disrupted BBB after TBI is known to release S100B into the serum. Therefore, it is recognized as a sensitive biomarker of TBI. However, in the study by Winter et al., serum S100B levels in the recruited patients with TBI were not correlated with the $K^{Trans}\;$values.

In 2018, Yoo et al. conducted a retrospective study in which they analyzed the MRI data of 44 patients with postconcussion syndrome (PCS) after mild TBI [14]. Mild TBI was defined as loss of consciousness for 0–30 min, posttraumatic amnesia or alteration of consciousness for less than 24 h. For comparison, 32 control patients with normal structural findings on brain MRI were selected from their database. Out of 44 TBI patients, 21 showed multifocal T2 hyperintense lesions in either the subcortical or deep white matter. Yoo et al. measured $K^{Trans}$ and $v_{e}$ values. The median $K^{Trans}$ and $v_{e}$ values at the dorsolateral midbrain and $K^{Trans}$ at bilateral frontal gray-white matter were significantly higher in mild TBI patients than in control patients. In addition, Yoo et al. evaluated the symptoms of PCS and cognitive impairment using the Rivermead postconcussion symptoms questionnaire and computerized neurocognitive function test. They found that delayed recall scores were significantly correlated with $v_{e}$ values at T2 hyperintense white matter lesions, but no other significant correlation was observed. In addition, the median $v_{e}$ value at predilection sites for diffusion axonal injury (i.e., bilateral frontal and temporal gray-white matter interfaces, corpus callosum (splenium), and dorsolateral midbrain) was significantly higher in TBI patients with abnormal performance in the forward distal span test than in patients with good performance. However, no other significant correlation or difference related to post-TBI clinical symptoms was observed.

In 2020, O’Keeffe et al. prospectively recruited five professional mixed martial arts (MMA) fighters and 19 adolescent rugby players [11]. They evaluated the DCE-MRI results and their correlation with biomechanical parameters in these individuals. In MMA fighters, DCE-MRI was examined prefight for a baseline and again within 120 h post competitive fight, and rugby players were assessed pre and postseason. BBB permeability maps were created using the slope of the contrast agent concentration in each voxel over time. Additionally, O’Keeffe et al. directly measured head impact using a mouthguard with an accelerometer and gyroscope. Moreover, brain tissue deformation was estimated by the finite element model, including the brain, skull, scalp, meninges, cerebrospinal fluid and 11 pairs of bridging veins. The authors found that BBB disruption observed in DCE-MRI in MMA fighters was correlated with the duration and repeatability of the strike. In rugby players, in DCE-MRI undertaken postseason, the BBB disruption was found in the periventricular regions in 10 of 19 players. O’Keeffe et al. also examined systemic biomarkers of BBB damage in rugby players. The level of brain-derived neurotrophic factor was significantly increased postseason, and levels of monocyte chemoattractant protein-1 and S100B were significantly increased postmatch. In addition, a weak positive correlation was observed between S100B levels and the degree of BBB disruption on DCE-MRI.

In 2021, Yoen et al. retrospectively investigated BBB disruption in 42 patients with mild TBI with PCS (loss of consciousness for 0–30 min and posttraumatic amnesia or alteration of consciousness for <24 h) using DCE-MRI [13]. For comparison, DCE-MRI data were collected from 29 controls. In their study, automatic whole-brain segmentation was used to select the regions of interest. Increased permeability due to mild TBI was shown in the Patlak model, but not in the extended Tofts and Kermode models. In the Patlak model, the mean $K^{Trans}\;$value in the bilateral cerebral cortex was significantly higher in mild TBI patients than in the controls. The mean $v_{p}$ values in the bilateral cerebellar white matter and brainstem were significantly lower in patients with mild TBI compared to the controls. Furthermore, the mean $K^{Trans}\;$value of the bilateral cerebral cortex was significantly higher in TBI patients who showed poor performance in the auditory continuous performance test.

## 4. Discussion

Here, we reviewed previous studies in which the usefulness of DCE-MRI for detecting BBB disruption following TBI was evaluated. The BBB disruption was measured with parameters such as $K^{Trans}$, $v_{e}$ and $v_{p}$.

In previous studies, following TBI, the values of $K^{Trans}\;$and $v_{e}$ were significantly increased, or showed a tendency to increase, in the areas in which the BBB was disrupted or at predilection sites for diffusion axonal injury [12,13,14]. In a study by Yoen et al., the $v_{p}$ value was found to be significantly lower in the cerebellar white matter and brainstem after mild TBI [13]. These alterations in DCE-MRI parameters ($K^{Trans}$, $v_{e}$ and $v_{p}$) in previous studies are indicative of BBB disruption following TBI. Additionally, O’Keeffe et al. used a linear fit model to demonstrate that the BBB disruption on DCE-MRI was caused by the repetitive strike to the head in five MMA fighters, and showed BBB disruption in the periventricular regions in 10 of 19 rugby players [11].

In clinical practice, the diagnosis of TBI, especially mild TBI, remains challenging. The two primary mechanisms of TBI include concussion and diffuse axonal injury [18,19]. These injuries cause microscopic lesions in the cerebral cortex and white matter. Even when a patient complains of persistent clinical symptoms after TBI, conventional imaging tools, such as brain MRI and computed tomography, frequently reveal no abnormal findings [18]. Therefore, there is an unmet need for accessible biomarkers that can accurately and reliably detect TBI. Diffusion tensor tractography has been reported to be useful for detecting microscopic neural tract injury in patients who had no abnormal findings on conventional MRI after TBI [20,21]. However, its use in clinical practice is limited because of frequently occurring false-positive and negative results [22]. The blood vessels run throughout the entire brain, and the BBB surrounds most of the blood vessels in the brain. A direct injury to the brain by contusion or diffuse axonal injury seems to induce an injury to the BBB in the cerebral cortex, periventricular area or brain stem. Furthermore, some previous studies reported that alterations in parameters that indicate BBB disruption following TBI are significantly correlated with poor cognitive performance [13,14]. We believe that the BBB disruption can indirectly implicate microscopic injury to the brain parenchyma due to trauma. In addition, disruption of the BBB causes secondary injury by allowing neurotoxins to enter the brain parenchyma without filtration [4]. Therefore, the findings of BBB disruption in DCE-MRI can be indicative of primary and secondary brain injury caused by TBI.

The methods used in the previous studies are relatively acceptable in that the authors included the patients with definite brain injury, and the DCE-MRI data were analyzed in an appropriate way. However, relatively small numbers of patients with TBI were involved in the previous studies, and sample size calculation was not performed prior to initiating each study. Furthermore, two prospective studies (Winter et al.’s and O’Keeffe et al.’s studies) were performed without a control group.

In the future, the degree of alteration of DCI-MRI parameters according to the severity of TBI should be evaluated. For the practical use of DCE-MRI in TBI patients, studies for evaluating reliability of each DCI-MRI parameter and determining the most appropriate pharmacokinetic model should also be conducted.

In conclusion, previous studies have shown altered levels of DCE-MRI parameters ($K^{Trans}$, $v_{e}$ and $v_{p}$) following TBI in vulnerable brain areas, including the brain cortex and the brain stem, indicating BBB disruption in patients with TBI. Therefore, our review supports utilizing DCE-MRI for evaluating BBB disruption following TBI. Moreover, this technique may provide indirect evidence for the presence of microscopic injury of the brain parenchyma. However, due to the small number of studies, we cannot definitively confirm the usefulness of DCE-MRI in clinical practice in this context. Consequently, more high-quality studies are required to fully evaluate the potential of DCE-MRI in detecting BBC disruption in patients with TBI.

## Figures and Tables

**Table 1:** Summary of the included studies.

First Author, Year	Study Design	Number of Patients (E/C)	Parameters for Analysis	Summary of the Outcome
Winter, 2015 [12]	Single-arm prospective study	14 (7 mild, 4 moderate, and 3 severe TBI)	$K^{Trans}$	Although there was no statistical significance, as TBI severity became more severe, the $K^{Trans}$ value increased. $K^{Trans}$ value was significantly correlated with the SPECT findings.
Yoo, 2019 [14]	Retrospective study	44 (mild TBI)/32	$K^{Trans}$, $v_{e}$	$K^{Trans}$ and $v_{e}$ values at dorsolateral midbrain and $K^{Trans}$ at bilateral frontal gray-white matter were significantly higher in the TBI patients. Delayed recall scores were significantly correlated with $v_{e}$ values.
O’Keeffe, 2020 [11]	Single-arm prospective study	5 professional MMA fighters and 19 adolescent rugby players	Slope of contrast agent concentration	MMA fighters: the degree of BBB disruption was correlated with the duration and repeatability of the strike. Rugby players: BBB disruption was found in the periventricular regions.
Yoen, 2021 [13]	Retrospective study	42 (mild TBI)/29	$K^{Trans}$, $v_{p}$	$\mathrm{The}\;K^{Trans}\;$value in the bilateral cerebral cortex was significantly higher in mild TBI patients. $v_{p}$ values in the bilateral cerebellar white matter and brainstem were significantly lower in mild TBI patients.

E, experimental group; C, control group; TBI, traumatic brain injury; SPECT, single-photon emission computed tomography; BBB, blood brain barrier; MMA, mixed martial arts; $K^{Trans}$, volume transfer constant; $v_{e}$, extravascular extracellular space volume fraction; $v_{p}$, blood plasma volume fraction.

**Table 2:** Pharmacokinetic models utilized in each study.

	Pharmacokinetic Model	Parameters	Study
Tofts	$C_{t}\left(t\right)=K^{Trans}\int_{0}^{t}C_{p}\left(\tau \right)e^{\frac{K^{Trans}}{v_{e}}\left(\tau -t\right)}d\tau$	$K^{Trans}$, $v_{e}$	Winter (2015), Yoo (2018) [12,14]
Extended Tofts	$C_{t}\left(t\right)=v_{p}C_{p}\left(t\right)+K^{Trans}\int_{0}^{t}C_{p}\left(\tau \right)e^{\frac{K^{Trans}}{v_{e}}\left(\tau -t\right)}d\tau$	$K^{Trans}$, $v_{p}$, $v_{e}$	Yoen (2021) [13]
Patlak	$C_{t}\left(t\right)=v_{p}C_{p}\left(t\right)+K^{Trans}\int_{0}^{t}C_{p}\left(\tau \right)d\tau$	$K^{Trans}$, $v_{p}$	Yoen (2021) [13]

$C_{t}$, tracer concentration in tissue; $K^{Trans}$, volume transfer constant; $C_{p}$, tracer concentration in blood plasma; $v_{e}$, extravascular extracellular space (EES) volume fraction; $v_{p}$, blood plasma volume fraction.

## Data Availability

Not applicable.
